# Snapin, Positive Regulator of Stimulation- Induced Ca^2+^ Release through RyR, Is Necessary for HIV-1 Replication in T Cells

**DOI:** 10.1371/journal.pone.0075297

**Published:** 2013-10-10

**Authors:** Shigemi M. Kinoshita, Amane Kogure, Shizuka Taguchi, Garry P. Nolan

**Affiliations:** 1 Department of Pathology, Anatomy and Cell Biology, Thomas Jefferson University, Philadelphia, Pennsylvania, United States of America; 2 Laboratory of Immune Regulation, Osaka University Graduate School of Frontier Biosciences, Suita, Osaka, Japan; 3 Department of Molecular Pharmacology, Stanford University School of Medicine, Stanford, California, United States of America; 4 Department of Microbiology and Immunology, Stanford University School of Medicine, Stanford, California, United States of America; Salute San Raffaele University School of Medicine, Italy

## Abstract

To identify critical host factors necessary for human immunodeficiency virus 1 (HIV-1) replication, large libraries of short-peptide-aptamers were expressed retrovirally. The target of one inhibitor peptide, Pep80, identified in this screen was determined to be Snapin, a protein associated with the soluble N-ethyl maleimide sensitive factor adaptor protein receptor (SNARE) complex that is critical for calcium-dependent exocytosis during neurotransmission. Pep80 inhibited Ca^2+^ release from intracellular stores and blocked downstream signaling by direct interruption of the association between Snapin and an intracellular calcium release channel, the ryanodine receptor (RyR). NFAT signaling was preferentially abolished by Pep80. Expression of Snapin overcame Pep80-mediated inhibition of Ca^2+^/NFAT signaling and HIV-1 replication. Furthermore, Snapin induced HIV-1 replication in primary CD4^+^ T cells. Thus, through its interaction with RyR, Snapin is a critical regulator of Ca^2+^ signaling and T cell activation. Use of the genetically selected intracellular aptamer inhibitors allowed us to define unique mechanisms important to HIV-1 replication and T cell biology.

## Introduction

T cell activation is essential for productive HIV-1 infection in primary T cells since essential processes or molecules that permit HIV-1 replication become readily available following T cell induction [1], [2], [3]. This activation process is initiated by the interaction of the T cell antigen receptor (TCR) with antigen-derived peptide bound to the major histocompatibility complex (MHC) on the antigen presenting cells (APC). This cell-cell interaction activates signaling cascades and leads to the activation of NFAT, NF-*κ*B, and AP-1, which are involved in regulation of gene expression important for T cell proliferation and differentiation.

Calcium is critical for functional immune responses in T cells [4]. The interaction of antigen/MHC with TCR triggers TCR engagement and subsequent Ca^2+^ release from intracellular stores, such as the endoplasmic reticulum (ER), through inositol 1,4,5 trisphosphate receptor (IP3R) and RyR on the ER membrane. IP3R is opened by IP3 (inositol 1,4,5 trisphosphate), which is produced by TCR engagement [5], [6]. RyR is operated by a second messenger, cyclic ADP-ribose (cADPR); its concentration levels increase after T cell activation through TCR [7]. The depletion of ER Ca^2+^ stores activates Ca^2+^ release-activated Ca^2+^ (CRAC) channels in the plasma membrane and induces Ca^2+^ influx. The resulting, long-lasting elevation in cytoplasmic Ca^2+^ concentration activates Ca^2+^ signaling pathways, including those mediated by calcineurin and NFAT, that control T cell activation and differentiation in the immune response [8].

In this report, we used a dominant effector genetic selection scheme to select an aptamer, Pep80, that inhibits HIV-1 replication and T cell activation. The target of Pep80 was identified as Snapin, a synaptosomal-associated protein 25 kDa (SNAP-25) binding protein. The interaction of SNAP-25 and Ca^2+^ sensor synaptotagmin-I (Syt-I) is crucial for the release of dense core vesicles and sensitization of the SNARE complex during synaptic vesicle exocytosis. Snapin is involved in the modulation of the interaction between SNAP-25 and Syt-I necessary for the early synaptic vesicle docking. Snapin is therefore involved in the neurotransmitter release process and was originally thought to be exclusively expressed in neurons and located on synaptic vesicle membranes [9]. Later, it was demonstrated that Snapin is broadly distributed in many tissues [10] and interacts with various molecules such as regulators of G-protein signaling 7, type VI adenylyl cyclase, dysbindin-1, casein kinase 1*δ*, granulocyte colony-stimulating factor receptor, RyRs, Exo70, *α*_iA_-adrenoceptor (*α*_iA_-AR), transient receptor potential canonical 6 (TRPC6), and others [11], [12], [13]. A study of Snapin knock-out mice demonstrated that Snapin is involved in calcium-dependent, SNARE-mediated exocytosis and plays an important role in neurosecretion [14].

Here we report a novel function of Snapin in immune cells detected using a specific inhibitor aptamer peptide: Snapin regulates Ca^2+^ release from the calcium stores, such as the ER, by direct interaction with the intracellular calcium release channel, RyR, resulting in activation of Ca^2+^-dependent signaling pathways required for T cell activation. This peptide inhibitor allowed us to uncover previously unknown functions of Snapin in calcium regulation and HIV-1 replication in T cells.

## Results

### Selection of intracellular aptamers that inhibit HIV-1 transcription through NFAT, but not AP-1, signaling

We developed a dominant effector genetic screen for trans-acting peptides that act upon T cell signaling processes important to HIV-1 replication. Libraries (>10^7^ different members) of short peptides (10-mers) were expressed using a retroviral system [15]. We used Jurkat HIV-LTR *dipA* cells that had been transfected with a *dipA* gene driven by the HIV-1 promoter for this screen. These cells are killed by stimuli that activate HIV-1 long terminal repeat (LTR) activity such as phytohaemagglutinin (PHA) or tumor necrosis factor-alpha (TNF-α) [16]. Cells from the Jurkat HIV-LTR *dipA* line survive despite PHA treatment if an expressed peptide blocks signaling that normally leads to HIV-1 promoter activation [15].

Jurkat HIV-LTR *dipA* cells were infected with the retrovirus peptide library. One week after retrovirus transduction, the cells were stimulated with PHA. This stimulation was repeated six times at regular intervals over two months. After the sixth PHA stimulation, we isolated the GFP-positive (peptide-expressing) cells by flow cytometry. We prepared total cellular DNA from these surviving cells. Using specific primers, we rescued peptide-encoding inserts by PCR and subcloned them into the pBMN-IRES-GFP retrovirus vector. These retroviral supernatants were transduced into fresh Jurkat HIV-LTR *dipA* cells as in the original screen. This verified that several clones, including that encoding Pep80, were capable of inhibiting HIV-1 transcription.

**Figure 1:**
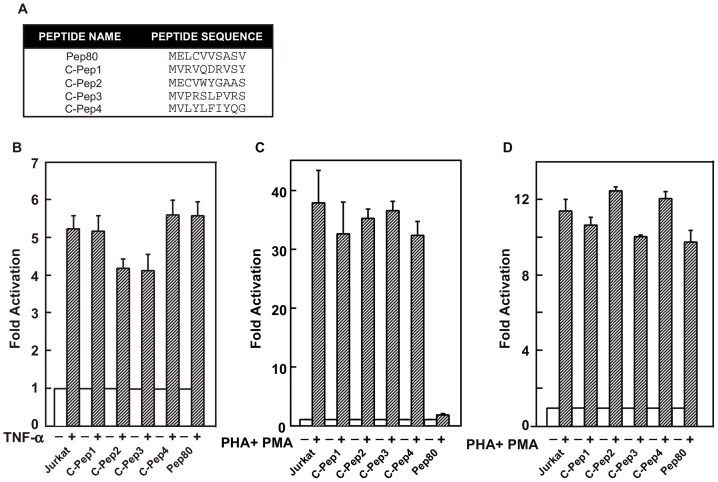
Pep80 preferentially inhibits the NFAT signaling pathway. (A) The sequences of control peptides and Pep80. (B–D) Luciferase reporter plasmids (B) p55-IgκLuc, (C) NFAT Luc, and (D) AP-1 were transfected into Jurkat cells with pBMN lacZ as the internal control plasmid. Cells were treated for 3 hr (8 hr for AP-1) with or without indicated agents (2 µg/ml PHA, 10 ng/ml PMA, and 10 ng/ml TNF-α) prior to measurement of luciferase activity. The experiments were repeated three times, and the results are plotted with error bars; values shown are the average ± SE. Reporter plasmid-transfected cells without treatment were assigned a value of 1 and used to calculate the fold activation. Transfection efficiencies were normalized to activity of a co-transfected lacZ plasmid.

To begin to understand how these peptides interfered with the T cell activation processes, we examined signaling pathways influenced by Pep80. The most critical cis-regulatory elements in HIV-1 LTR are the *κ*B regulatory elements that can be activated by NF-*κ*B or NFAT [17], [18], [19]. Pep80, as well as four control peptide clones that were picked from the library at random (Figure 1A), were individually transduced into Jurkat cells using retroviral delivery, and cells that expressed GFP (co-expressed with the peptide) were selected by flow cytometry. After culturing the selected cells, greater than 98% of cells were positive for GFP. Luciferase reporter plasmids driven either by three tandemly repeated NFAT binding sites from the IL-2 gene or by three tandemly repeated NF-*κ*B binding sites from the Ig*κ* chain were transfected into cells expressing peptides. With TNF-*α* stimulation we observed comparable luciferase induction of the NF-*κ*B-specific reporter plasmid in cells expressing the control peptides and Pep80 (Figure 1B). With PHA plus PMA stimulation, we observed the expected stimulation of the NFAT-specific reporter plasmid in cells expressing the control peptides. In cells expressing Pep80, NFAT-driven induction was completely inhibited (Figure 1C).

We then examined the effect of Pep80 on the AP-1 signaling pathway. AP-1 associates with the Rel domain of NFAT, and the holoprotein complex is critical for T cell activation [20], [21]. An AP-1-specific reporter plasmid (driven by five tandemly repeated AP-1 binding sites) was transfected into Jurkat cells and Jurkat cells expressing either control peptides or Pep80. With PHA plus PMA stimulation, levels of transcriptional activity were similar in Jurkat cells, Jurkat cells expressing control peptides, and Jurkat cells expressing Pep80 (Figure 1D). These results indicate that the inhibition of the NFAT signaling pathway by Pep80 does not involve inhibition of AP-1 activation; this was expected based on earlier work that indicated that AP-1 is a fundamental part of the NFAT holoprotein complex [20], [21]. Pep80 thus specifically inhibits the NFAT signaling pathway and the target host-cell molecule of Pep80 is a critical factor in the NFAT-specific signaling pathway.

### Aptamer inhibition of HIV-1 replication

**Figure 2:**
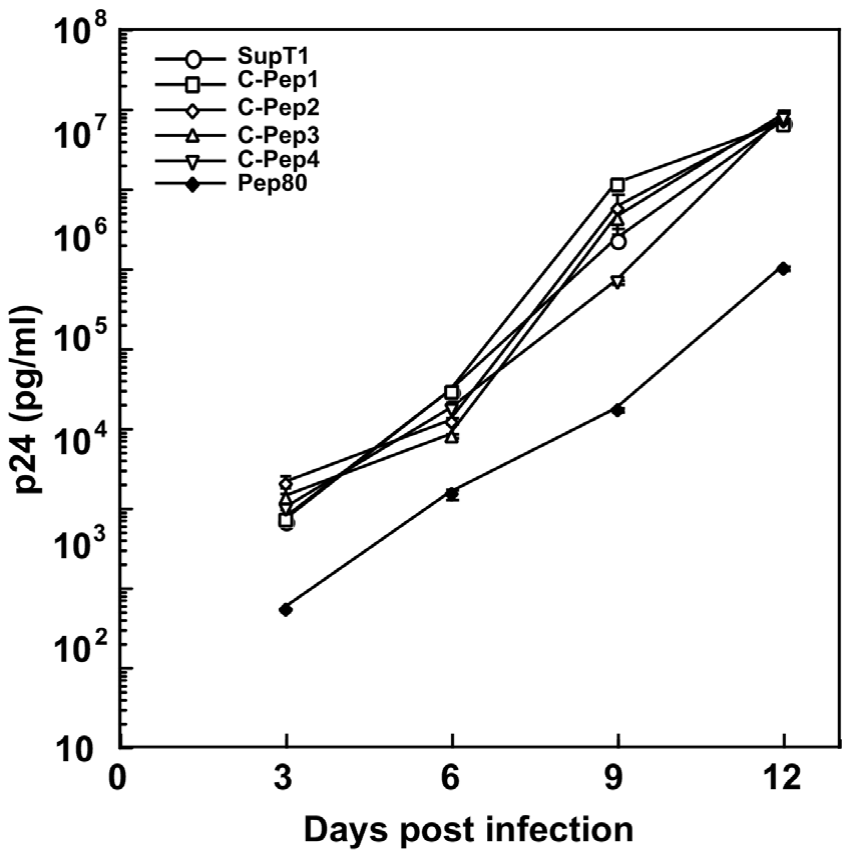
Pep80 strongly inhibits HIV-1 replication in T cells. SupT1 cells expressing one of four control peptides or Pep80 were challenged with HIV-1 (NL4-3) at a dose of 400 TCID_50_ per 5×10^4^ cells. P24^gag^ levels in culture supernatants were assayed from four wells on the indicated days after infection. P24^gag^ levels were normalized for cell number using an XTT assay. Data are presented as the average ± SE per 10^6^ cells. Similar results were observed in three independent experiments.

The screen was devised to select inhibitors of early events in T cell activation on which HIV-1 depends for its replication. We therefore examined whether Pep80 influenced HIV-1 replication in T cells. We prepared SupT1 clones expressing either control peptides or Pep80. SupT1 cells and SupT1 peptide-expressing cells were challenged by the HIV-1 T-tropic strain NL4-3. Over a time course of 12 days post HIV-1 challenge, HIV-1 replication was measured using a p24 ELISA. The HIV-1 replication level was similar in SupT1 cells and in those cells expressing the control peptides. In cells expressing Pep80, however, HIV-1 replication was strongly inhibited at every time point analysed over the time course of 12 days (Figure 2). This shows that Pep80 inhibits HIV-1 replication in T cells and suggests that the target molecule of Pep80 might be involved in HIV-1 replication.

### Snapin is the target of Pep80

To characterize the mechanism by which Pep80 acts, we identified the target molecule in host cells using a standard yeast two-hybrid screen. Pep80 was cloned into a yeast two-hybrid vector and a leukocyte-derived cDNA library was screened. Of eight cDNAs selected, four encoded a protein called Snapin. Other cDNAs selected encoded IL-2 receptor, L37 ribosomal protein, Gemin2, and Zn-15 related zinc finger protein.

**Figure 3:**
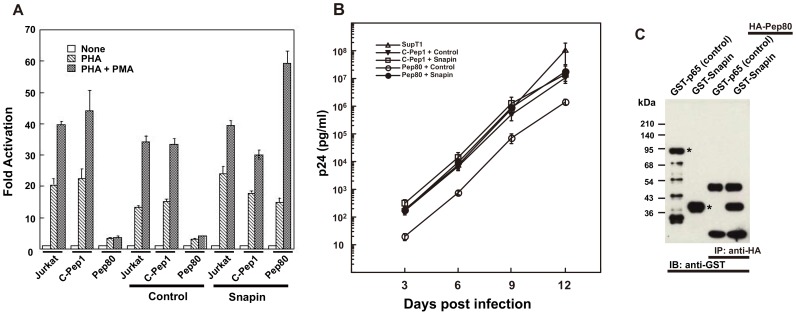
Snapin overcomes the inhibition of NFAT transcription and HIV-1 replication resulting from expression of Pep80. (A) NFAT Luc reporter plasmids were transfected into Jurkat cells expressing the indicated peptide with pBMN lacZ as the internal control plasmid. Cells were treated for 3 hr with or without indicated agents (2 *µ*g/ml PHA and 10 ng/ml PMA) prior to measurement of luciferase activity. The experiments were repeated three times; values shown are the average ± SE. Untreated Jurkat cells were assigned a value of 1 and data from these cells were used to calculate the fold activation. Transfection efficiencies were normalized to a co-transfected lacZ plasmid. (B)'pBMN-control IRES-Lyt2*α*' or pBMN-Snapin IRES-Lyt2*α*' retrovirus vectors were transduced into SupT1 cells expressing C-Pep1 or Pep80. These cells were challenged with HIV-1 (NL4-3) at a dose 400 TCID_50_ per 5×10^4^ cells. P24^gag^ levels in culture supernatants were assayed from four wells on the indicated days after infection. P24^gag^ levels were normalized to cell number determined using an XTT assay. Data are presented as the average ± SE per 10^6^ cells. Similar results were observed in three independent experiments. (C) 293T cells were co-transfected with the indicated combinations of expression vectors: HA-Pep80, GST-p65 (control), or GST-Snapin. Cell lysates were immunoprecipitated with anti-HA mAb and immunoblotted with anti-GST mAb. Purified GST-p65 (control) and GST-Snapin are shown as controls and marked with asterisks.

To examine whether Snapin overcomes Pep80 inhibition of the NFAT signaling pathway, we first performed a luciferase reporter assay using the NFAT-specific reporter plasmid. The NFAT-specific reporter was transduced into C-Pep1- or Pep80-expressing Jurkat cells with or without Snapin. With PHA plus PMA stimulation, NFAT-driven induction was significantly inhibited in cells expressing Pep80; however, cells expressing both Pep80 and Snapin showed luciferase levels similar to those of cells expressing the control peptide C-Pep1 and Snapin (Figure 3A). We also prepared SupT1 cells that expressed Snapin constitutively in the presence or absence of Pep80. When challenged with HIV-1 (NL4-3), Snapin expression alone did not alter HIV-1 replication in SupT1 cells compared to control cells. However, the inhibition of HIV-1 replication by Pep80 was completely overcome by Snapin expression (Figure 3B). From these experiments we concluded that Snapin is a target of Pep80. These experiments also indicate that Snapin is involved in NFAT signaling and is necessary for HIV-1 replication.

To determine whether Pep80 specifically binds to Snapin, we carried out affinity binding experiments using Snapin fused to glutathione-S-transferase (GST-Snapin) and an HA-tagged Pep80 (HA-Pep80). GST-Snapin and HA-Pep80 were co-transfected into 293T cells, and cell lysates were immunoprecipitated with anti-HA antibody. Western blotting of the lysates with anti-GST antibody showed that Snapin specifically associated with Pep80 (Figure 3C). We confirmed that Pep80 does not bind to GST by using GST-p65 fusion protein as a negative control (Figure 3C). This experiment also ruled out the possibility of non-specific binding between Snapin and the Gal4 DNA-binding domain that can result in a false positive in the Gal-4-based yeast two-hybrid system.

### Snapin localizes to the ER membrane in T cells

**Figure 4:**
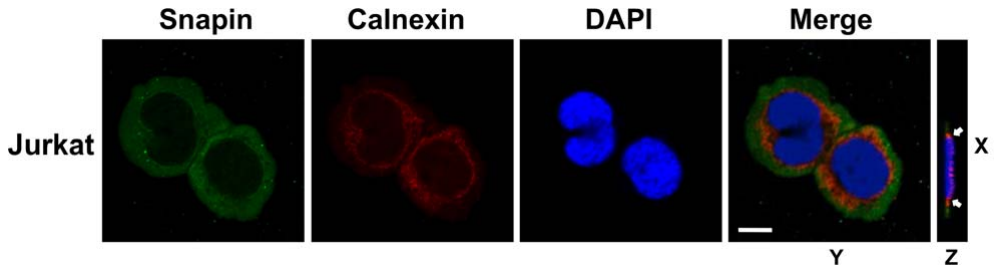
Snapin interacts with RyR in T cells. Jurkat cells were immunostained with anti-Snapin antibody and anti-calnexin antibody. Cells were then examined by confocal microscopy. Z; Reconstructed three-dimensional structures of Jurkat cells based on Z-stack analysis of immunostained Snapin (green) and calnexin (red). Nuclei were stained with DAPI (blue). White arrows indicate co-localization. Bar, 10 *µ*m.

To examine the expression and localization of Snapin in T cells, we performed confocal microscopy using an anti-Snapin antibody. Staining surrounded the nucleus and was localized in cytoplasm in Jurkat T cells (Figure 4). It was previously shown that Snapin interacts with fragments of RyR 1, RyR 2, or RyR3 *in vitro* [12], [13]. We hypothesized that Snapin interacts with RyR and regulates Ca^2+^ signal in T cells. To examine this hypothesis, we first tested whether Snapin was expressed on or near the ER membrane where RyR is mainly located. We performed confocal microscopy using an anti-calnexin antibody. Calnexin is an ER-resident chaperone protein, which is used as an ER membrane marker [22]. The merged data for Snapin (green) and calnexin (red) showed significant overlap (orange) (Figure 4). This result suggests that Snapin and RyR co-localize and therefore might interact in T cells on the ER membrane.

### Pep80 inhibits the interaction between Snapin and RyR in T cells

**Figure 5:**
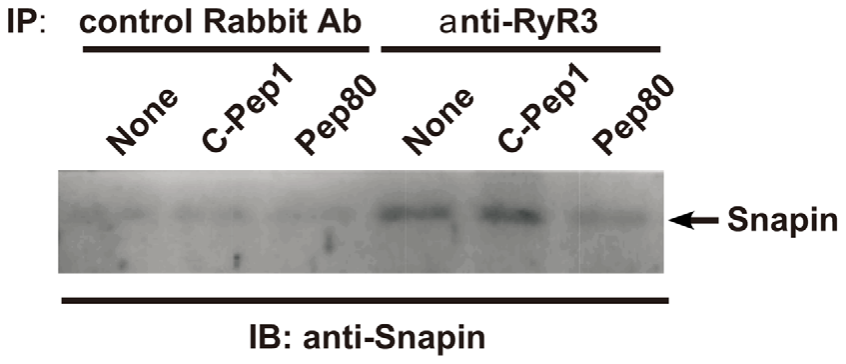
Pep80 inhibits the interaction between Snapin and RyR in T cells. After crosslinking the ER fraction from Jurkat cells and from C-Pep-1- or Pep80-expressing Jurkat cells, samples were immunopreciptated with anti-RyR3 antibody and immunoblotted with anti-Snapin antibody.

Next we examined the physical interaction between endogenous Snapin and RyR with or without Pep80 in Jurkat cells. We prepared an ER fraction from either C-Pep1- or Pep80-expressing Jurkat cells, immunoprecipitated using anti-RyR3 antibody or control rabbit antibody, and immunoblotted with an anti-Snapin antibody. In the control antibody immunoprecipitate, we detected a very narrow band in all cells. When an anti-RyR3 antibody was used for immunoprecipitation, we observed a clear band from Jurkat cells and from C-Pep1-expressing Jurkat cells but not in Pep80-expressing Jurkat cells (Figure 5). The band in Pep80-expressing Jurkat cells was observed at a similar level when we used control rabbit antibody. This indicates that Snapin physically interacts with RyR in T cells and that the Snapin inhibitor peptide Pep80 disrupts this interaction. This suggests the possibilities that Snapin regulates Ca^2+^ release from intracellular stores by interaction with RyR and that this regulation is critical for NFAT signaling pathway and T cell activation.

### Snapin regulates Ca^2+^ channel function of RyRs

RyRs regulate Ca^2+^ release from intracellular stores such as the ER by TCR/CD3-mediated stimulation [7]. Our screen showed that Snapin was activated by PHA, which mimics T cell activation through TCR. We also demonstrated a physical interaction between Snapin and RyR (Figure 5). We therefore considered the possibility that Snapin regulates Ca^2+^ release from intracellular stores through RyRs after T cell activation by TCR/CD3-mediated stimulation. By using the calcium-sensing dye indo-1-AM, the Ca^2+^ concentration in the cytoplasm of cells can be measured, and the use of Ca^2+^-free medium with EGTA allows measurement of Ca^2+^ release from intracellular stores [23]. We also performed the experiment with medium containing Ca^2+^ to measure Ca^2+^ influx as described below.

**Figure 6:**
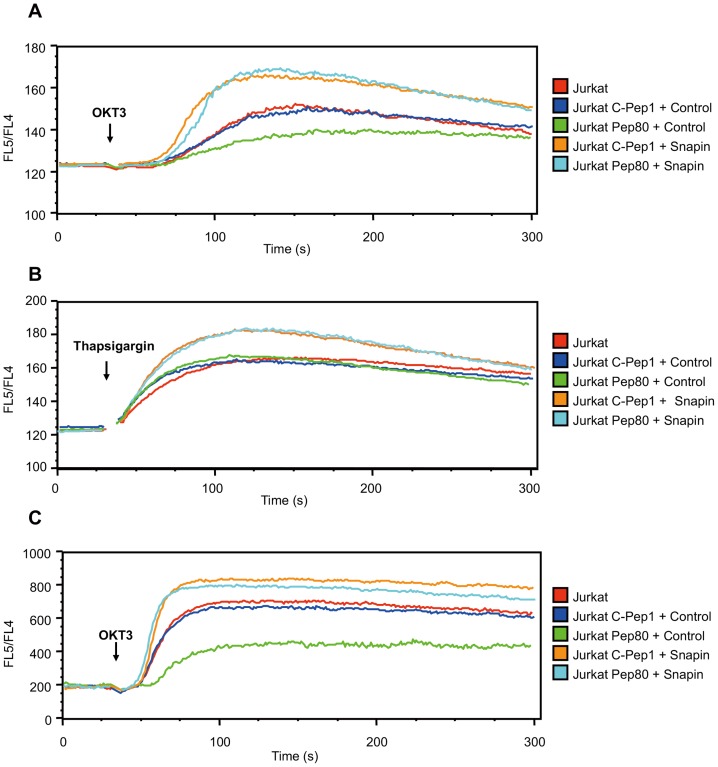
Snapin regulates Ca^2+^ efflux and influx in T cells. (A, B) Indicated cells were suspended in Ca^2+^-free medium containing 10 mM EGTA. “Control” indicates control retrovirus, whereas “Snapin” indicates that cells were infected with Snapin-encoding retrovirus. Cells were transduced with either Pep80 or C-Pep1. Cells were stained with APC-anti-Lyt2*α*' and were loaded with indo-1-AM calcium sensor dye. EGTA was added, and after 30 s (A) OKT3 or (B) thapsigargin was added. The FL5/FL4 ratio (400 nm/510 nm fluorescence emission) was monitored using a flow cytometer. (C) Cells were suspended in medium containing Ca^2+^. After 30 s, OKT3 was added. The FL5/FL4 ratio was monitored using a flow cytometer.

Cells were suspended in Ca^2+^-free medium containing 10 mM EGTA to avoid Ca^2+^ influx from outside the cells. We took a non-stimulated baseline reading for 30 seconds, and then cells were stimulated with anti-CD3 antibody OTK3. We observed similar intracellular Ca^2+^ concentrations after OKT3 stimulation in Jurkat cells and cells expressing the control peptide C-Pep1. However, the intracellular Ca^2+^ concentration was significantly lower in Pep80-expressing Jurkat cells after stimulation (Figure 6A). These data show that the inhibition of the interaction between Snapin and RyR by Pep80 blocks Ca^2+^ release from intracellular stores.

When we over-expressed Snapin in cells expressing either C-Pep1 or Pep80, the intracellular Ca^2+^ concentration was elevated relative to cells that expressed either peptide or a control plasmid that did not express Snapin (Figure 6A). These data indicate that Snapin is the target molecule for Pep80 and that Snapin is involved in Ca^2+^ release from intracellular stores. Snapin expression likely caused calcium-induced calcium release (CICR) through RyR.

To confirm that the inhibition of Ca^2+^ release from intracellular stores by Pep80 depends on TCR/CD3-mediated signaling, we examined whether Pep80 was involved in Ca^2+^ store depletion by thapsigargin treatment. Thapsigargin is an inhibitor of sarcoplasmic/endoplasmic reticulum Ca^2+^ ATPase (SERCA) and causes artificial depletion of calcium stores by inhibiting Ca^2+^ transport into the ER independently of intracellular Ca^2+^ release channels [24]. We did not observe a significant difference in the intracellular Ca^2+^ concentration after thapsigargin stimulation in Jurkat cells or Jurkat cells expressing either C-Pep-1 or Pep80 (Figure 6B). As Pep80 did not inhibit calcium depletion upon thapsigargin stimulation, Snapin only regulates Ca^2+^ release from intracellular stores upon TCR-mediated signaling through intracellular Ca^2+^ release channels and does not influence SERCA-related Ca^2+^ store depletion. However, we observed that Ca^2+^ release from intracellular stores after thapsigargin treatment was distinctly enhanced in Jurkat cells that over-expressed Snapin and either C-Pep-1 or Pep80 when compared to Jurkat cells or to Jurkat cells expressing a control vector with either C-Pep-1 or Pep80 (Figure 6B). As Snapin expression induced CICR after thapsigargin treatment, CICR through RyR requires Snapin, and Snapin activation is induced by TCR/CD3-mediated stimulation. Our data rule out induction of CICR by Snapin though IP3R since thapsigargin treatment increases the cytoplasmic Ca^2+^ concentration but does not induce production of IP3 [6], [24], which is necessary for Ca^2+^ release from intracellular stores through IP3R.

We next examined whether Snapin is involved in Ca^2+^ influx in T cells. We measured the intracellular Ca^2+^ concentration in indo-1-loaded cells expressing either C-Pep1 or Pep80 as described above. In this case, cells were suspended in medium containing Ca^2+^. The level of Ca^2+^ influx was similar between Jurkat cells and Jurkat cells expressing C-Pep1. Expression of Pep80 inhibited Ca^2+^ influx compared with cells that expressed C-Pep1, and this inhibition by Pep80 was reversed when Snapin was also expressed (Figure 6C). This indicates that inhibition of Snapin activity by Pep80 blocks Ca^2+^ efflux from intracellular stores and influences Ca^2+^ influx and confirms that Snapin is the target of Pep80.

### Snapin knockdown inhibits Ca^2+^ influx in T cells

**Figure 7:**
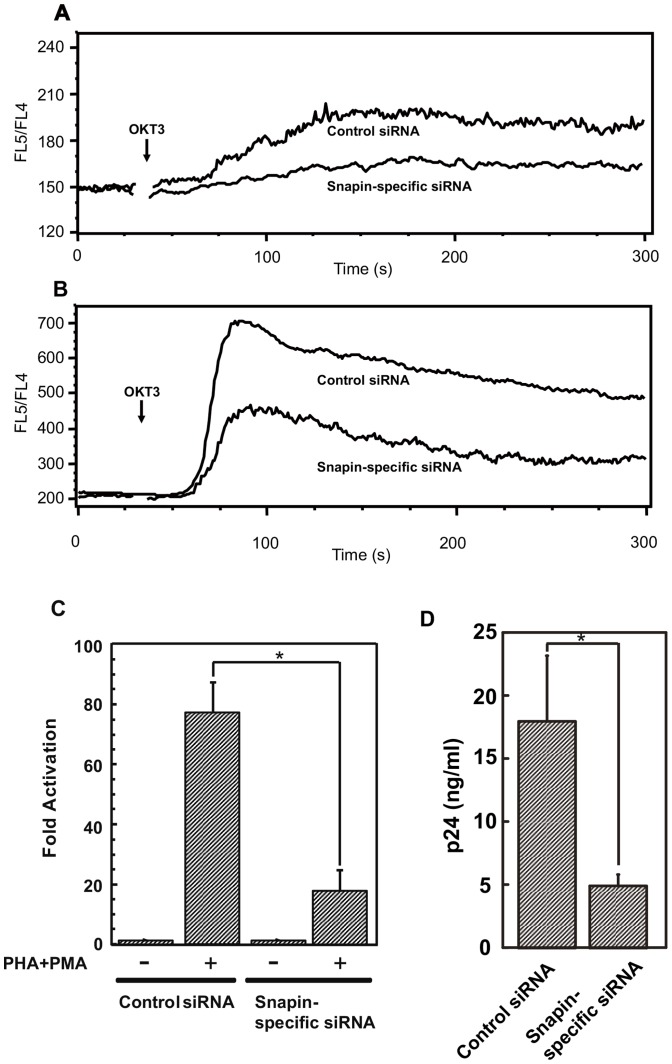
siRNA-mediated knockdown of Snapin inhibits Ca^2+^ influx and HIV replication. Jurkat cells that were transfected with Snapin-specific siRNA or control siRNA were suspended in (A) Ca^2+^-free medium with 10 mM EGTA or (B) Ca^2+^-containing medium. After 30 s, OKT3 was added. The FL5/FL4 ratio was monitored using a flow cytometer. (C) NFAT Luc reporter plasmid and pBMN lacZ were transfected into Jurkat cells that were transfected with Snapin-specific siRNA or control siRNA. Cells were treated for 3 hr with or without PHA and PMA prior to measurement of luciferase activity. The experiments were repeated three times; values shown are the average ± SE. Data from cells without treatment were assigned a value of 1 and were used to calculate the fold activation. Transfection efficiencies were normalized to a co-transfected lacZ plasmid. (D) SupT1 cells that were transfected with Snapin-specific siRNA or control siRNA were challenged with replication incompetent HIV-1-Ea. P24^gag^ levels in culture supernatants were assayed from four wells 48 hr after HIV-1 challenge. P24^gag^ levels were normalized for cell number using XTT assay. Data are presented as the average ± SE per 10^6^ cells. Similar results were observed in three independent experiments. * indicates *p*<0.05 by *t* test.

To elucidate the function of Snapin in T cell biology, we introduced a Snapin-specific small interfering RNA (siRNA) or a control siRNA into Jurkat cells using the AMAXA nucleofector apparatus. Real-time PCR was used to evaluate Snapin expression 24 hr and 48 hr after siRNA transduction. The level of *Snapin* mRNA was decreased by about 70% by the Snapin-specific siRNA in comparison with levels in cells transfected with control siRNA. To examine the effects of Snapin knockdown on Ca^2+^ efflux from intracellular stores, the intracellular Ca^2+^ concentration was measured using indo-1-loaded cells as described above. The OKT3-induced intracellular Ca^2+^ release was inhibited by the knockdown of Snapin (Figure 7A), indicating that Snapin was involved in Ca^2+^ release from intracellular stores in T cells. We also examined whether Snapin regulates Ca^2+^ influx in indo-1 loaded T cells by flow cytometry. Snapin knockdown blocked OKT3-induced Ca^2+^ influx (Figure 7B). Thus, Snapin is an important player in Ca^2+^ release from intracellular stores; Snapin appears to operate through RyR to open the CRAC channel and allow Ca^2+^ influx into T cells.

After Ca^2+^ influx, NFAT is dephosphorylated by calcineurin, which is activated by Ca^2+^, and is rapidly translocated into the nucleus where it activates the gene expression program for T cell activation [25]. To examine whether Snapin regulates NFAT gene transcription, we transduced luciferase reporter plasmids driven by three tandemly repeated NFAT binding sites into Jurkat cells transduced with either Snapin-specific siRNA or control siRNA. After PHA plus PMA stimulation, NFAT-specific transcription activity was observed in control cells but was inhibited in cells treated with Snapin-specific siRNA (Figure 7C). Thus, the inhibition of Ca^2+^ influx by knockdown of Snapin expression blocks downstream NFAT activation. These data demonstrate that Snapin plays a crucial role in induction of NFAT-regulated transcription.

### Snapin regulates Ca^2+^ signaling critical to HIV replication

To test whether Snapin is involved in HIV-1 replication, we carried out single-round HIV-1 infection using replication incompetent HIV-1 (HIV-1-Ea); this virus has a retrovirus ampho-tropic envelope instead of the wild-type HIV-1 envelope. We transduced Snapin-specific siRNA or control siRNA into SupT1 cells 24 hr before HIV-1-Ea challenge. We measured HIV-1 replication by p24 ELISA 48 hr after the challenge. HIV-1 replication was blocked by the Snapin-specific siRNA (Figure 7D). This demonstrates that Snapin is also critical for HIV-1 replication in T cells.

**Figure 8:**
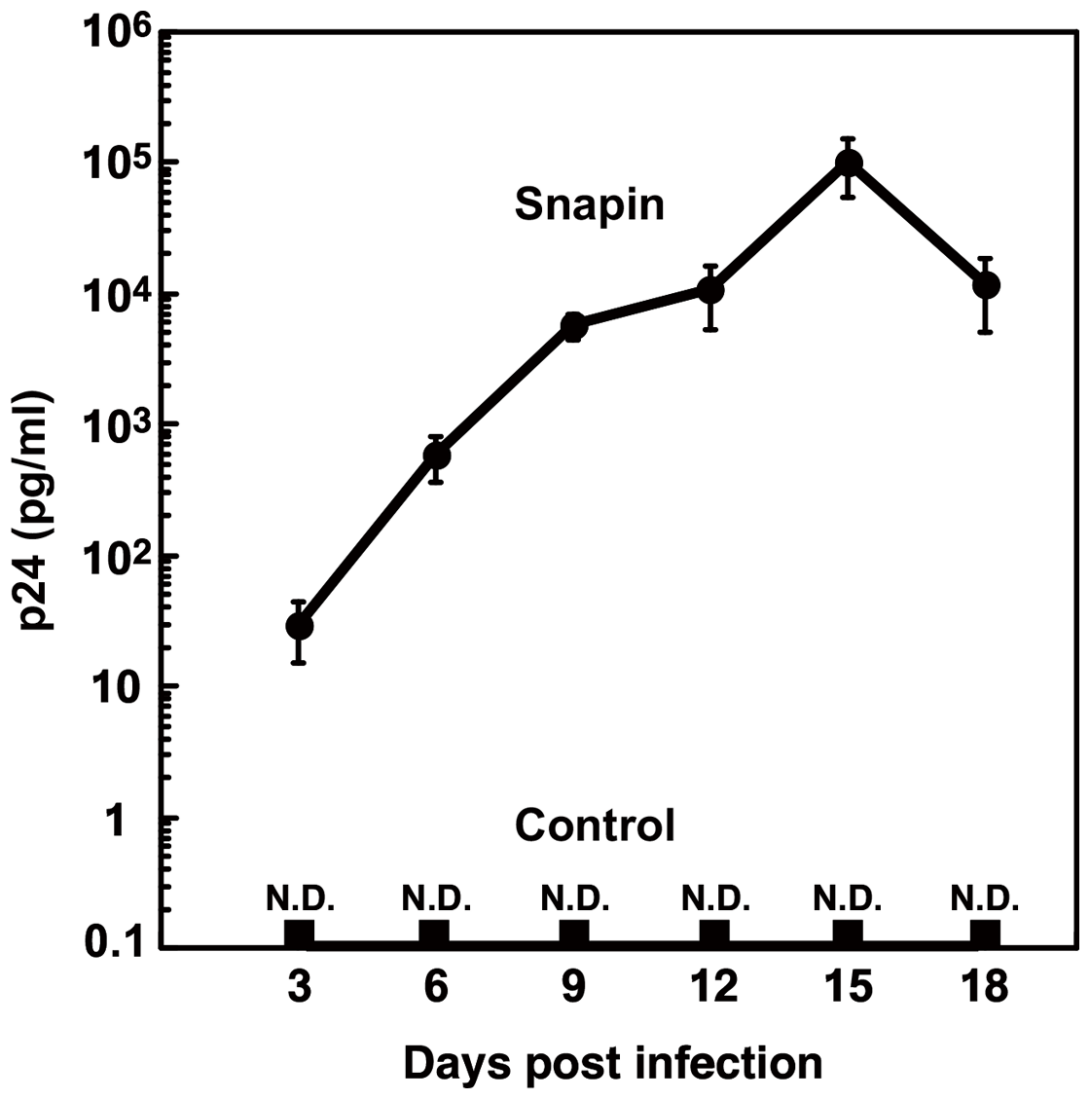
Snapin induces HIV-1 replication in primary CD4^+^ T cells. pBMN-control-IRES-Lyt2*α*' and pBMN-Snapin-IRES-Lyt2α' retrovirus vectors were transduced into human primary CD4^+^ T cells. CD4^+^ T cells were challenged by HIV-1 (NL4-3) at 400 TCID_50_ per 1×10^5^ cells. P24^gag^ levels in culture supernatants were assayed from four wells on the indicated days after infection. P24^gag^ levels were normalized for cell number using an XTT assay. Data are presented as the average ± SE per 10^6^ cells. Similar results were observed in three independent experiments. N.D.; not detected.

Finally, we examined HIV-1 replication in primary CD4^+^ T cells with or without ectopic Snapin expression. Snapin-expressing or control retrovirus was transduced into primary CD4^+^ T cells. HIV-1 (NL4-3) was applied to these CD4^+^ T cells, and HIV-1 replication levels were measured by a p24 ELISA. We did not observe HIV-1 replication in control retrovirus-transduced primary CD4^+^ T cells. However, in primary CD4^+^ T cells that expressed Snapin, HIV-1 replication was dramatically induced without exogenous activation (Figure 8). This shows that HIV-1 replication was induced by Snapin expression in primary CD4^+^ T cells. Thus, Snapin is necessary for T cell activation; activation, in turn, is required for HIV-1 replication in primary CD4^+^ T cells. We conclude that Snapin, which is induced by TCR-mediated activation, facilitates Ca^2+^ release from intracellular stores by operating RyR and positively regulates Ca^2+^ signaling important to T cell activation and HIV-1 replication.

## Discussion

For productive HIV-1 infection in primary T cells there is a requirement for intracellular signaling pathway activation [1], [2], [3]. Such signaling induces reverse transcription, nuclear translocation, integration, and transcription from the HIV-1 promoter [1], [2], [3], [18], [19]. Interestingly, unlike replication in primary T cells, HIV-1 replication occurs promiscuously in many CD4^+^ T cell lines in the absence of exogenous stimulation. It is likely that critical host factors for HIV-1 replication that are primed during physiological activation of primary T cells are constitutively active in these T cell lines [26]. By determining the nature of the differences that result in HIV-1 replication in certain cells, critical features of the biology of HIV-1 and potential therapeutic modalities will be revealed.

We used a genetic screening approach to create peptidic aptamers that act as biological modifiers, termed dominant effectors, to block HIV-1 access to host factors required for effective replication. The system was designed to allow the identified peptide inhibitors to be used as tools to isolate the host factors upon which they act. The dominant effector peptide described here, Pep80, repressed host pathways that are important for HIV-1 replication in T cell lines. Using this intracellular genetic selection system we identified Snapin as a host factor that regulates HIV-1 replication in T cells. We showed that Snapin is involved in Ca^2+^ signaling necessary for T cell activation and HIV-1 replication using the Snapin-specific inhibitor Pep80 and also by Snapin knockdown experiments.

Ca^2+^ is an important second messenger that controls various physiological functions [27], [28]. All these processes begin by induction of Ca^2+^ release from intracellular Ca^2+^ stores such as the ER and the sarcoplasmic reticulum (SR). Upon T cell activation, Ca^2+^ is released after IP3 binding to the IP3R or after induction of second messenger cADPR for RyRs [5], [6], [7]. Our data indicated that Pep80 blocks OKT3-mediated store depletion through intracellular Ca^2+^ release channels (Figure 6A) but that Pep80 does not influence the inhibition of Ca^2+^ influx into the ER by thapsigargin through SERCA (Figure 6B). We also demonstrated that Snapin directly associates with RyR; this association was interrupted by Pep80 in Jurkat cells (Figure 5). This shows that the inhibition of OKT3-mediated store depletion by Pep80 depends on the interruption of association between RyR and Snapin. These findings indicate that Snapin is a positive regulator that controls Ca^2+^ release from intracellular stores through RyR by TCR/CD3-mediated stimulation in T cells.

Moreover, we observed an increase in Ca^2+^ release from intracellular stores upon thapsigargin treatment when Snapin was over-expressed (Figure 6B). Although thapsigargin treatment in the absence of Snapin increased the cytoplasmic Ca^2+^ concentration, CICR through RyR did not occur since thapsigargin-induced Ca^2+^ store depletion was not inhibited by Pep80. When Snapin was over-expressed and cells were treated with thapsigargin, CICR was observed (Figure 6B). This shows that Snapin function is induced by TCR/CD3-mediated stimulation and is necessary for CICR. IP3R and RyR are known to affect CICR differently [6], [29]. For IP3R, Ca^2+^ is necessary, but not sufficient [30]. IP3R-mediated CICR requires IP3. However, thapsigargin treatment does not induce IP3. In contrast to IP3R, CICR through RyR is induced by Ca^2+^ alone [31]. This indicates that in Snapin-expressing cells after thapsigargin treatment CICR occurs through RyR. Our data clearly show that Snapin is necessary for induction of CICR through RyR and that the association between Snapin and RyR regulates Ca^2+^ release from intracellular stores and ensuing T cell activation and HIV-1 replication. It has been reported that Snapin interacts with both α_iA_-AR and TRPC6 and facilitates Ca^2+^ influx through the TRPC6 channel after α_iA_-AR activation in PC12 cells [11]. In this case, Snapin interacts with TRPC6 as a receptor-operated Ca^2+^ channel and regulates α_iA_-AR-operated Ca^2+^influx. Snapin regulates both Ca^2+^ release from intracellular stores and Ca^2+^influx through different Ca^2+^ channels such as RyR and TRPC6. There is no obvious homology between RyR and TRPC6. Therefore, how Snapin regulates different Ca^2+^ channels remains unclear.

Snapin had not previously been implicated in HIV-1 replication and T cell activation. Snapin is small: 15 kDa and only 136 amino acids. Despite this fact, Snapin interacts with a number of molecules to regulate their functions [12], [13]. Snapin binds to the fragments from all three isoforms of RyRs [13]. Snapin interacts with the fragment of RyR2 C-terminal region (4596–4752) containing a single predicted cytosolic loop and a stretch of long hydrophobic segment that has been proposed to form a putative transmembrane domain, M7 (4718–4750) [13]. The binding region for Snapin on RyR is near the channel pore and ion filter (4820–4829) [32]. Mutation of RyR1 or RyR2 causes functional disorders of receptors and induces malignant hyperthermia, central core disease (CCD), catecholaminergic polymorphic ventricular tachycardia, and arrhythmogenic right ventricular dysplasia type 2. These disorders are linked to the mutations of the RyR C-terminal region [33]. For example, mutations of RyR1 C-terminal region (3916–4973) are often observed in CCD [34], and mutations of RyR2 C-terminal region (3778–4959) cause inherited ventricular tachycardia [32]. We demonstrated that the interaction between Snapin and RyR is important for Ca^2+^ release from intracellular stores and ensuing Ca^2+^ signaling in T cells using Pep80. It is possible that Snapin cannot bind to the C-terminal region in the mutants of RyRs and cannot, therefore, regulate Ca^2+^ efflux from intracellular stores and CICR. More detailed analysis of the binding between RyRs and Snapin will be required to determine whether the inability of RyRs to bind Snapin underlies these disorders.

RyR3 is expressed in many tissues and in T cells and is abundant in brain, especially hippocampus, corpus striatum, and diencephalons. It has been reported that Ca^2+^ dysregulation via the amplification of CICR through RyR is involved in Alzheimer's disease, dementia, and brain aging [35], [36], [37]. This amplified CICR through RyR leads to apoptosis, necrotic cell death, and finally massive cell death in brain. In fact, CICR through RyR in the hippocampus increases during brain aging [38]; however, the levels of RyR expression do not change in brain during aging in normal rats [39]. This suggests that certain regulatory molecules that control the function of RyR, such as Snapin, could lead to the amplification of CICR through RyR during aging. The dysregulation of Snapin during aging might be related to brain dysfunction. We demonstrate that Snapin is necessary for induction of CICR through RyR in T cells. Snapin might play a similar role in brain. Our Snapin-specific inhibitor peptide Pep80 should prove useful in elucidation of the mechanism of Ca^2+^ dysregulation in aging brains and Alzheimer's disease.

The expression of Pep80 inhibited Ca^2+^ release from intracellular stores by TCR/CD3-mediated OKT3 stimulation and preferentially blocked the downstream signaling through of NFAT (Figures 1C and 6A). The long-lasting Ca^2+^ release from intracellular stores occurs through RyR; this Ca^2+^ release activates CRAC channels and leads to long-lasting Ca^2+^ influx responsible for NFAT signaling during T cell activation and proliferation [7], [8]. We demonstrated that Ca^2+^ release from intracellular stores through RyR is regulated by interaction between Snapin and RyR and that this Ca^2+^ release is crucial for NFAT transcription in T cells. Our results using Pep80 clearly showed that Snapin plays a critical role in Ca^2+^ signal-dependent T cell activation by operating RyR to release Ca^2+^ from intracellular stores and induce CICR.

The dominant effector peptide system we developed creates artificial specific inhibitors of targeted signaling molecules. The selected peptide inhibitors can be used to elucidate the physiological function of their targets. Here we described selection of peptides that interfere with T cell activation-dependent processes that support HIV-1 replication. Using the peptide selected, we identified the host factor involved in HIV-replication and elucidated a novel function of this host factor, Snapin, in Ca^2+^ release from intracellular stores that is critical for T cell activation and HIV-1 replication.

## Materials and Methods

### Plasmid construction

The sequences of C-Pep1 through C-Pep4 and Pep80 were inserted into pBMN-IRES-GFP using BamH I and Sal I sites. The coding sequence of Snapin was inserted into pBMN-IRES-Lyt2*α*' between BamH I and Sal I sites. pEBG-Snapin (GST-Snapin) was constructed by inserting the cording sequence of the Snapin into pEBG. The construction of retrovirus peptide library was described previously [15].

### Screening of peptide library and rescue of survivor peptides

Recombinant retroviruses representing the peptide library were prepared using the Phoenix Ampho retrovirus packaging cell line and infected into 3×10^8^ Jurkat HIV-LTR dipA cells as described previously [15], [18]. The cells infected with the peptide library were stimulated with 2 *µ*g/ml PHA six times over a period of 2 months. During this selection period, cells were cultured in RPMI 1640 containing 2.5% fetal calf serum (FCS). After this selection, GFP-positive living cells were sorted by flow cytometry, and total cellular DNA was prepared from these cells using the QIAamp Blood Kit (QIAGEN). The DNA was amplified by PCR using the primers 5′-AGCTAGATCGCAGTGTGCCACCATG-3′ and 5′-CTCGAGTCAGTCAGTCA-3′. The PCR fragment was ligated into pBMN-IRES-GFP and this DNA was transformed into STBL4 electrocompetent *E. coli* cells (Invitrogen) according to the manufacturer's protocol.

### Luciferase assay

Jurkat cells and peptide-expressing Jurkat cells were cultured in RPMI 1640 containing 10% FCS. The luciferase assay was performed as described previously [18]. Cells (1×10^6^) were transfected with 1 *µ*g of reporter plasmid, 1 *µ*g of indicated plasmids, and 1 *µ*g pBMN LacZ by FuGENE6 according to the manufacturer's protocol (Roche Applied Science). Thirty-six hours after transfection, cells were treated as indicated and cell extracts were measured for luciferase activity. LacZ activity was assayed by standard methods.

### HIV infection and p24 assay

SupT1 cells and SupT1 cells expressing indicated peptides were cultured in RPMI 1640 medium containing 2.5% FCS. HIV-1 infection and p24 assay were performed as described previously [19]. Cells were infected with HIV-1 by incubating cells with NL4-3 (for SupT1 cells, 400 TCID_50_/5×10^4^ cells; for primary CD4+ T cells; 400 TCID_50_/1×10^5^ cells) in 0.5 mL of culture medium at 37°C for 4 hr. After HIV-1 challenge, cells were washed with culture medium and plated in five wells in a 48-well plate (for SupT1 cells, 5×10^4^/well; for primary CD4+ T cells, 1×10^5^ cells). Virus replication was measured on the indicated days after HIV-1 challenge by p24 ELISA according to the manufacturer's protocol (ZeptoMetrix Corporation). P24_gag_ levels were normalized for cell number measured using the XTT assay.

### Yeast two-hybrid screening

Pep80 was fused in-frame to the Gal4 DNA binding domain through a three-glycine spacer in pAS2-1 GAL4 plasmid. A pACT2-human leukocyte library (Clontech) was used for the screening by the yeast two-hybrid method in Y190 yeast cells as described [40] with minor modifications (40 mM 3-aminotriazole was added to medium).

### Immunoprecipitation and immunoblotting

To probe the interaction between Snapin and Pep80, 293T cells were seeded at 1.5×10^6^ cells per 60-mm dish in DMEM containing 10% FCS. Twenty-four hours later, cells were transfected with 3 µg of indicated plasmids (GST-p65, GST-Snapin, and HA-Pep80) using the calcium phosphate co-precipitation technique [18]. Immunoprecipitation and immunoblotting were performed as described previously [26]. Two days later, the cells were lysed for 20 min in ice in buffer containing 10 mM CHAPS, 50 mM NaCl, 20 mM Tris (pH 7.5) and cleared by centrifugation. The lysates were immunoprecipitated with anti-HA mAb (clone HA-7, Sigma) and immunoblotted with anti-GST mAb (G1160, Sigma) and anti-mouse-HRP conjugate (A9044, Sigma). Blots were visualized with ECL Plus Western Blotting Detection System (GE Healthcare). GST fusion proteins were purified over a glutathione S-transferase column according to the manufacturer's protocol (Pharmacia).

To detect the interactions between Snapin and RyR3 or Pep80, Jurkat cells and indicated peptide-expressing Jurkat cells were cultured in RPMI 1640 containing 5% FCS. After crosslinking by dithiobis succinimidyl propionate (22585, Pierce), ER purification was performed with the Endoplasmic Reticulum Isolation Kit (ER0100, Sigma) according to the manufacturer's instructions. ER fractions were lysed in RIPA buffer (20 mM Tris-HCl, 150 mM NaCl, 1% NP-40, 1% deoxycholate, 0.1% sodium dodecyl sulfate (SDS)) containing protease inhibitors (1860932, Thermo). Lysates were pre-cleared 1 hour with Protein G beads (17-0618-01, GE Healthcare), and then incubated 2 hour with protein G beads coupling with anti-human RyR3 antibody (ab9082, Millipore) or control antibody. The beads were washed six times with RIPA Buffer and were boiled 5 min with sample buffer (50 mM Tris-HCl, pH 6.8, 2% SDS, 1% bromophenol blue, 10% glycerol, 5% β-mercaptoethanol). Eluted proteins were resolved by SDS-PAGE, and western blot analysis was performed using anti-Snapin antibody (148 002, Synaptic Systems) as primary antibody and anti-rabbit IgG-HRP (18-8816, eBioscience) as secondary antibody. Blots were visualized with ECL Plus Western Blotting Detection System (NEL103001EA, Perkin Elmer).

### Laser scanning confocal microscopy

Cytospin preparations of Jurkat or SupT1 cells were prepared by centrifugation of 100 µl of cell suspension (5×10^5^ cells/ml) at 450 rpm for 7 min in a Shandon Cytospin 2 cytocentrifuge (Shandon Southern Products Ltd.). The slides were air dried for 20 min and fixed with 4% paraformadehyde for 15 min at room temperature and were permeabilized in 90% methanol for 15 minutes on ice. Slides were washed (PBS with 1% BSA) to block background staining and were incubated with 10% donkey serum in PBS for 1 hr. Slides were washed once in wash buffer. Snapin was detected using a primary rabbit polyclonal anti-Snapin antibody (148002, Synaptic Systems) and a DyLight™488-conjugated secondary anti-rabbit (711-485-152, Jackson ImmunoResearch). Calnexin was detected using a primary mouse monoclonal antibody (SC-80645, Santa Cruz Biotechnology) and a DyLightTM594-conjugated secondary anti-mouse antibody (715-515-151, Jackson ImmunoResearch). Antibody stains were performed at 4°C for 1 hr. Slides were washed three times in wash buffer. The slides were stained with DAPI and were coverslipped with Prolong Gold Antifade reagent (Molecular Probes, Invitrogen).

Cells were visualized by sequential laser excitation using a Nikon laser scanning confocal microscope (ECLIPSE Ti) and an oil immersion 60× objective. Images were acquired using ImageJ (NIH) software and compiled using Adobe Illustrator CS2. Z-stacks for three-dimensional reconstructions were collected at 0.25 µm z axis interval and 6.0 µm total height with a 60× objective. Three-dimensional structures of cells stained with Snapin, calnexin, and DAPI were constructed from Z-stacked data (ImageJ).

### Intracellular calcium measurements using flow cytometry

The cells were stained with an anti-mouse CD8α antibody conjugated to PE (PharMingen, 01045B). After cell-surface staining, the cells were loaded with indo-1-AM (0.3 *µ*M) in RPMI1640 complete medium containing 0.025% pluronic F-125 and 4 mM probenecid and incubated at 37°C for 30 min. After the first incubation, the cells were washed and then incubated at 37°C for 30 min in OPTI MEM. After the second incubation, the cells were washed three times to remove any free dye and were resuspended in PBS without Ca^2+^; 1% BSA and 10 mM ethylene glycol tetraacetic acid (EGTA) were added before flow cytometry analysis for measurement Ca^2+^ efflux from intracellular stores. HBSS with 1% BSA was added for measurement of Ca^2+^ influx to approximately 1×10^6^ cells/ml. The cytoplasmic Ca^2+^ was determined by measurement of the 400 nm/510 nm ratio using a flow cytometer. The baseline fluorescence of untreated cells was measured for 30 s, the tube was removed to add 5 *µ*g/ml OKT3 (eBioscience, 16-0037) or 1 *µ*M thapsigargin (Sigma, T9033), and then flow cytometry analysis was continued.

### siRNA-mediated knockdown of Snapin

The *Snapin* sequence targeted was 5′-GACUGAGACGGCUAAACCdAdTdT-3′ and the scrambled control sequence was 5′-CUAAGGCGAACCGUAAACdGdTdT-3′. siRNAs were obtained from Sigma and were transfected into T cell lines using an AMAXA nucleofector apparatus (program U-014, 3 *µ*g of siRNA per 1×10^7^ cells).
